# Harmine Inhibits Multiple TLR-Induced Inflammatory Expression through Modulation of NF-κB p65, JNK, and STAT1

**DOI:** 10.3390/life12122022

**Published:** 2022-12-03

**Authors:** So-Jung Jin, Youngju Song, Hong Shik Park, Kye Won Park, SeungGwan Lee, Hee Kang

**Affiliations:** 1 Department of Horticultural Biotechnology, College of Life Sciences, Kyung Hee University, Yongin 17104, Republic of Korea; 2 Department of Biomedical Science and Technology, Graduate School, Kyung Hee University, Seoul 02447, Republic of Korea; 3 Department of Physical Education, Kyung Hee University, Yongin 17104, Republic of Korea; 4 Department of Food Science and Biotechnology, Sungkyunkwan University, Suwon 16419, Republic of Korea; 5 Humanitas College, Kyung Hee University, Yongin 17104, Republic of Korea

**Keywords:** harmine, macrophages, TLR, inflammation, iNOS, TNF-α, NF-κB, JNK, AP-1, STAT1

## Abstract

Harmine is a beta-carboline alkaloid present in various plants, including in the seeds of *Peganum harmala* L. This study aimed to investigate the anti-inflammatory activity and mechanism of harmine using macrophages stimulated with various toll-like receptor (TLR) agonists and a model of endotoxemia. The expression of inflammatory mediators induced by ligands of TLRs 2, 3, 4, and 9 were examined in thioglycollate-elicited peritoneal macrophages isolated from BALB/c and C57BL/6 mouse strains. Further, the activation of NF-κB, MAPK, AP-1, and STAT1 was explored using lipopolysaccharide (LPS) and polyinosinic:polycytidylic acid (poly(I:C)). Finally, the liver inflammatory response during endotoxemia was examined. Harmine inhibited inducible nitric oxide synthase, cyclooxygenase-2 (COX-2), tumor necrosis factor-α (TNF-α), interleukin-6 (IL-6), IL-12, and other markers induced by various TLR agonists. The inhibition of NF-κB activity by harmine occurred via the modulation of p65 phosphorylation, independent of IκBα degradation. The inhibition of AP-1 activity by harmine was associated with the modulation of JNK. Harmine inhibited the LPS-induced serine and tyrosine phosphorylation of STAT1, but only affected serine phosphorylation by poly(I:C) treatment. In vivo, harmine inhibited iNOS and COX-2 expression during endotoxemia. Collectively, the results show that harmine can be effective against infectious inflammation through modulation of NF-κB, JNK, and STAT1.

## 1. Introduction

As the population of aging individuals is increasing, the number of people with acute or chronic inflammation is increasing. Although inflammation itself is necessary to recover from infection and tissue damage, it is potentially detrimental to the host cells and thus should be tightly regulated. In the context of excessive inflammatory responses in which multiple immune cells are involved, monocytes and T cells are mobilized to the site. In particular, these recruited monocytes replace tissue-resident macrophages and differentiate into activated macrophages, exacerbating tissue damage [1]. Therefore, the screening of most anti-inflammatory drug candidates or phytochemicals targets the activity of resident or differentiated macrophages. 

The key strategy in the search of anti-inflammatory agents is to understand the molecular events induced by toll-like receptors (TLRs) in activated macrophages, regardless of being tissue-resident or monocyte-derived. TLRs are expressed mainly by immune cells and, to some extent, non-immune cells, and are well-defined receptors for pathogen-associated molecular patterns. Generally, it is recognized that TLRs 1,2, 4, 5, 6, and 10 are expressed on the cell surface, detecting ligands from bacteria or fungi, whereas TLRs 3, 7, 8, 9, 11, 12, and 13 are located in endosomes, most of them sensing nucleic acids from microbes, including viruses [2]. However, this division is not always clear-cut. For example, TLR2 and TLR4 can bind to severe acute respiratory syndrome coronavirus 2 (SARS-CoV-2) structural proteins [3]. In addition to these infectious entities, TLRs can detect host-derived damage-associated molecular patterns (DAMPs) [4].

After binding to their ligands, TLRs recruit the adaptor molecules MyD88 or TRIF to initiate the signal transduction pathways that terminate in the activation of NF-κB, activator protein 1 (AP-1), and interferon (IFN) regulatory factors [5]. Such transcription factors are responsible for inducing the expression of vasoactive and microbicidal enzymes such as cyclooxygenase-2 (COX-2) and inducible nitric oxide synthase (iNOS), and inflammatory and antiviral cytokines such as tumor necrosis factor-α (TNF-α), interleukin-1β (IL-1β), IL-6, IL-12, and type I IFN (α/β) [4,6,7,8]. Some of these cytokines again activate their producer cells and neighboring cells in an autocrine and paracrine manner. Particularly, signal transducers and activation of transcription-1 (STAT-1) is an important transcription factor necessary for IFN-mediated responses. When type I IFN and other IFN members such as type II IFN (γ), bind to their respective receptors, the receptor-associated janus kinases (JAKs) become active and phosphorylate STAT1 on tyrosine residue, which induces formation of homodimers or heterodimers [9]. These complexes migrate to the nucleus and bind to their target genes, acting in concert with NF-κB [10]. In addition to phosphorylation of tyrosine, serine phosphorylation enables STAT1 to enter the nucleus and exert effects on transcription [10]. It remains elusive as to which kinases are specific for serine phosphorylation. Moreover, there is a crosstalk between the TLR and the JAK/STAT pathways. TLR2, TLR4, and TLR9 can directly induce STAT1 phosphorylation at the serine independent of TLR-induced type I IFN expression [10]. 

Harmine (7-methoxy-1-9H-pyrido[3,4-b]-indole) is a beta-carboline alkaloid present in various plants that were first isolated from the seeds of *Peganum harmala* L., which grows in the Middle East and China [11]. Plants containing this compound have been used in folk medicine to give a psychoactive reaction [11]. Pharmacological studies of harmine have revealed that it has neuroprotective, tremogenic, bone-protective, anti-diabetic, anti-angiogenic, anti-depressant, antiviral, and anti-inflammatory activities [12,13,14,15,16,17,18,19,20].

The target of the anti-inflammatory mechanism of harmine has been determined as NF-κB: harmine decreases TLR4-induced NF-κB transcriptional activity and TNF-α-induced p65 nuclear translocation in RAW 264.7 cells [19]. With respect to the canonical NF-κB pathway, cytosolic p65/p50, the major member of the NF-κB family, is maintained by IκBα, but the exposure of macrophages to TLR ligands or TNF-α activates IκB kinase to induce IκBα degradation, which allows cytosolic p65 to be phosphorylated and migrate to the nucleus [21]. It remains unclear at which point harmine inhibits NF-κB activity. Moreover, there may be more signaling molecules targeted by harmine. Specifically, whether harmine affects responses to other members of the TLR family needs to be determined. In this study, we used thioglycollate-elicited peritoneal macrophages isolated from BALB/c and C57BL/6 mouse strains, both of which have a different degree of macrophage activation [22,23]. These thioglycollate-elicited macrophages are differentiated from monocytes [24]. Using primary macrophages, we aimed to investigate the detailed anti-inflammatory effects of harmine on iNOS, COX-2, and pro-inflammatory cytokine responses induced by stimulation with TLR4 and other TLR ligands. Specifically, the activation of NF-κB, MAPK, AP-1, and STAT1 have been explored using the TLR4 agonist lipopolysaccharide (LPS) and the TLR3 agonist polyinosinic:polycytidylic acid (poly(I:C). Finally, the liver inflammatory response during endotoxemia was examined.

## 2. Materials and Methods

### 2.1. Animals

Seven-week-old male BALB/c mice or C57BL/6 mice were obtained from Koatech (Pyuntek, South Korea). The mice were housed in a temperature- and humidity-controlled pathogen-free animal facility with a 12 h light-dark cycle and fed a commercial diet, with water available ad libitum. All mice underwent a 1 week of adjustment prior to in vitro or in vivo experiments. For the in vivo experiment, mice were randomly assigned to a control or harmine group (n = 6). The animal protocol (KHUASP-18-005) was approved by our institutional committee, and mice were cared for in accordance with the US National Research Council for the Care and Use of Laboratory Animals specifications (1996).

### 2.2. Macrophage Isolation

The mice were injected intraperitoneally (i.p.) with 2 mL of 3.5% sterile thioglycollate solution (BD, Sparks, MD, USA), and sacrificed 4 days later via carbon dioxide inhalation. Peritoneal exudate cells were isolated by peritoneal lavage with cold DMEM (HyClone, Logan, UT, USA). After centrifugation, the cells were resuspended in DMEM containing 10% fetal bovine serum (FBS; HyClone) and 1% penicillin–streptomycin (WelGene, Kyungsan, Republic of Korea). The cells were plated overnight at 37 °C, and non-adherent cells were removed.

### 2.3. Cell Viability Assay

Cells (4 × 10^4^ cells) were seeded in 96-well plates overnight and left to adhere overnight. Then, the culture medium was removed and replaced with a fresh medium containing harmine (Sigma, St. Louis, MO, USA) for 24 h. Subsequently, the cell viability was determined using the 3-(4,5-dimethylthiazol-2-yl)-2,5-diphenyltetrazolium bromide (MTT) assay. Cell viability was expressed as a percentage relative to the control cells.

### 2.4. Stimulation of Cells with TLR Ligands

For RNA preparation and western blotting analysis, adherent cells (2 × 10^6^ cells) in 6-well plates were incubated with harmine for 12 h and then stimulated with 100 ng/mL LPS (serotype 055:B5, Sigma), 50 μg/mL poly(I:C) (Sigma), 10 μg/mL lipoteichoic acid (LTA) from Staphylococcus aureus (Sigma), or 2 μM phosphorothioate-modified CpG oligonucleotide(tccatgacgttcctgacgtt)(Integrated DNA Technologies, Coralville, IA, USA) for the indicated time shown in the figure legends. In some assays, 0.5 ng/mL IFN-γ (BD Pharmingen, San Diego, CA, USA) was used for priming. For supernatant collection, adherent cells (5 × 10^5^ cells) in 24-well plates were incubated with harmine for 12 h and then stimulated with 100 ng/mL LPS or poly(I:C) for the indicated time shown in the figure legends.

### 2.5. Western Blotting Analysis

Cells were rinsed in cold PBS and then lysed on ice in 0.1 mL of RIPA buffer (50 mM Tris-HCl, pH 7.5; 150 mM NaCl; 1 mM EDTA; 20 mM NaF; 0.5% NP-40; and 1% Triton X-100) containing a phosphatase inhibitor cocktail (Sigma) and a protease inhibitor cocktail (Quartett, Berlin, Germany). Protein concentrations were determined using the Bradford protein assay reagent (Bio-Rad), and the samples were diluted with sodium dodecyl sulfate (SDS) buffer and boiled for 3 min. The samples were separated on an 8% or 10% SDS-polyacrylamide gel and the separated proteins were transferred to polyvinylidene fluoride membranes. Nonspecific binding to the membranes was blocked by incubation in 5% skim milk in Tris-buffered saline with 0.1% Tween 20 (TBST) for 1 h. The membranes were incubated with COX-2 (Cayman, Ann Arbor, MI, USA), iNOS, IκBα, NF-κB p65, phospho-NF-κB p65 serine 276, phospho-JNK, JNK, phospho-ERK1/2, ERK, phospho-p38, p38, phospho-STAT1 serine 727, phospho-STAT1 tyrosine 701, STAT1, GAPDH, and lamin (Cell Signaling Technology, Beverly, MA, USA) diluted in 5% skim milk in TBST overnight at 4 °C. The blots were washed with TBST and incubated for 1 h with anti-rabbit horseradish peroxidase-conjugated antibodies. Immunoreactive bands were detected with EzWestLumi plus (ATTO, Tokyo, Japan) and analyzed using an EZ-Capture MG (ATTO). The band density of each protein was quantified using ImageJ software and normalized with GAPDH (Supplementary material Figure S2).

### 2.6. Cytokine Analysis

Supernatants harvested at 24 h after stimulation with LPS or poly(I:C) were analyzed using an enzyme-linked immunosorbent assay (ELISA). Cytokine concentration was determined using the IL-12p70, TNF-α, and IL-6 DuoSet ELISA kits (R&D Systems, Minneapolis, MN, USA).

### 2.7. Luciferase Assay

NF-κB transcriptional activity was measured using RAW 264.7 cells transfected with the pGL4.32 [luc2p/NF-κB-RE/Hygro] vector encoding the firefly luciferase reporter gene (luc2P) driven by five copies of an NF-κB response element (Promega, Madison, WI, USA). The transfected RAW 264.7 cells were plated into 96-well plates and left overnight. The medium was changed, and the cells were pretreated with harmine for 12 h and then stimulated with LPS or poly(I:C) for 6 h. Luciferase activity was measured using the Dual-Glo^®^ luciferase assay system (Promega).

### 2.8. Intraperitoneal Injection of LPS

C57BL/6 mice received i.p. injection of harmine or saline for 4 days and then i.p.. injection of 1 mg/ kg LPS. At 3 h after LPS injection, the mice were sacrificed through euthanasia and the liver was collected and frozen in nitrogen gas for further analysis.

### 2.9. RNA Isolation and Real-Time PCR

Total RNA was isolated using RNeasy Mini Kt (QIAGEN), and cDNA was reverse-transcribed using a High Capacity RNA-to-cDNA kit (Applied Biosystems, Foster, CA, USA). Diluted cDNA was mixed with Power SYBR Green PCR Master mix (Applied Biosystems) and 2 pmol of primers specific for each of iNOS, COX-2, TNF-α, or GAPDH. The amplification of cDNA was performed using a StepOnePlus real-time PCR system (Applied Biosystems). After initial heat denaturation at 95 °C for 10 min, PCR conditions were set at 95 °C for 15 s and 60 °C for 60 s for 40 cycles. For each PCR, a corresponding mRNA sample without reverse transcription was included as the negative control. The quantification of cDNA copy number was achieved using the comparative C_T_ method.

### 2.10. Statistical Analysis

Data were analyzed by Student’s t test or ANOVA followed by an LSD post-hoc test. Analyses were performed using IBM SPSS 22 software. All *p* values less than 0.05 were considered significant.

## 3. Results

### 3.1. Harmine Inhibited LPS-Induced Inflammatory Mediators in Mouse Peritoneal Macrophages

**Figure 1:**
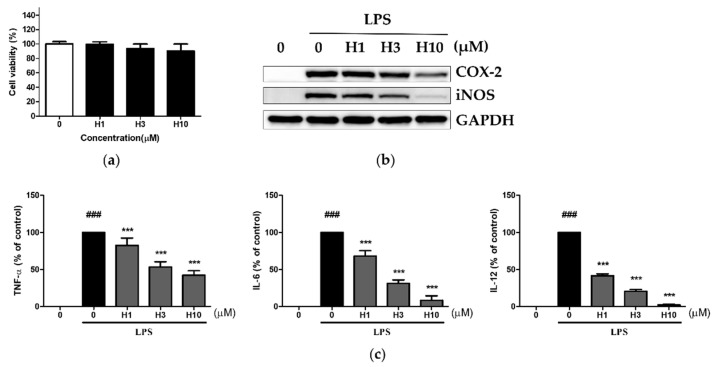
Harmine inhibits TLR4-triggered inflammatory mediators. Peritoneal macrophages were isolated from BALB/c mice and cultured with harmine. (**a**) The cell viability after 24 h of treatment was determined using the MTT assay and bars indicate the percentages relative to control cells (0 μM) (n = 3). (**b**) Peritoneal macrophages were pretreated with harmine at the indicated concentrations for 12 h and stimulated with LPS at 100 ng/mL for 24 h. Whole cell protein was extracted and the levels of COX-2 and iNOS were analyzed by western blotting using GAPDH as an internal control. A representative experiment of three independents experiments is shown. (**c**) Protein levels of IL-12, TNF-α, and IL-6 in the supernatant collected after 24 h of LPS stimulation were analyzed by ELISA (n = 3). ### *p* < 0.005 vs. LPS (–) control, *** *p* < 0.005 vs. LPS (+) control.

A literature search shows that harmine exhibits cytotoxic effects in various cancer cell lines, with a minimum IC50 of approximately 15 μM at 24 h [11]. Considering these previous findings, we limited the maximum concentration of harmine to 10 μM for the viability assay of macrophages. Using the MTT assay, harmine was not found to be toxic against peritoneal macrophages isolated from BALB/c mice (Figure 1a) and C57BL/6 mice (Figure S1). Thus, subsequent assays were conducted based on these ranges. Stimulation with LPS led to COX-2 and iNOS protein synthesis and harmine inhibited their levels in a dose-dependent manner (Figure 1b and Figure S2a). LPS induced the production of IL-12 (IL-12 p70), TNF-α, and IL-6 in the supernatant (Figure 1c). The levels of IL-12 decreased with the growing harmine concentrations, in a dose-dependent manner. Consistent with a previous report that used RAW 264.7 cells, reductions in TNF-α and IL-6 production were also observed [19]. We then examined whether the inhibition of these inflammatory mediators occurred at the level of the mRNA using C57BL/6 macrophages. Harmine decreased the expression of LPS-induced inflammatory marker genes, indicating that the anti-inflammatory activity of harmine occurs at the transcript level (Figure S3).

### 3.2. Harmine Inhibited Poly(I:C)-Induced Inflammatory Mediators

**Figure 2:**
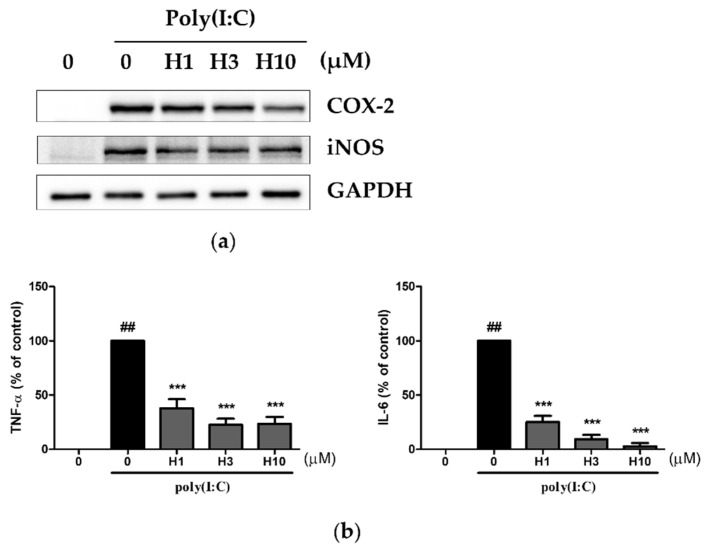
Harmine inhibits TLR3-induced inflammatory expression in peritoneal macrophages. Peritoneal macrophages from BALB/c mice were cultured with harmine for 12 h and then stimulated with poly(I:C) at 50 μg/mL for 24 h. (**a**) Whole cell protein was extracted and the levels of COX-2 and iNOS were analyzed by western blotting using GAPDH as an internal control. A representative experiment of three independent experiments is shown. (**b**) Protein levels of TNF-α and IL-6 in the supernatant collected after 24 h of poly(I:C) stimulation were analyzed by ELISA (n = 3). ## *p* < 0.01 vs. poly(I:C) (–) control, *** *p* < 0.005 vs. poly(I:C) (+) control.

**Figure 3:**
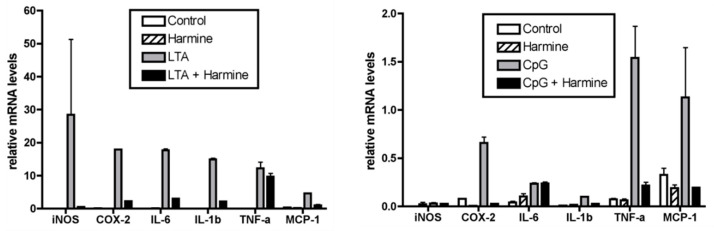
Harmine inhibits the TLR2- or TLR9-induced inflammatory responses. Peritoneal macrophages from C57BL/6 mice were cultured with harmine for 12 h and stimulated with lipoteichoic acid (LTA) from Staphylococcus aureus (10 μg/mL) or phosphorothioate-modified CpG oligonucleotide (2 μM) for 6 h. Gene expression was analyzed using realtime RT-PCR (n = 2).

Poly(I:C), a synthetic analog of double-stranded RNA was used to trigger the TLR3-induced inflammatory response using peritoneal macrophages isolated from BALB/c mice. As with LPS, stimulation with poly(I:C) induced iNOS and COX-2 protein expression (Figure 2a and Figure S2b). These protein bands were slightly inhibited by the harmine treatment. With regard to inflammatory cytokines, there was a detectable amount of TNF-a and IL-6, but not IL-12, in the supernatant (Figure 2b). The secretion of TNF-α and IL-6 was potently suppressed by the harmine treatment. We also examined whether such inhibition applied to other TLR agonists. The TLR2 agonist, lipoteichoic acid (LTA), and the TLR9 agonist, CpG DNA, were used to stimulate peritoneal macrophages isolated from the C57BL/6 mice. Harmine decreased the LTA-induced gene expression of iNOS, COX-2, IL-1β, IL-6, TNF-α, and monocyte chemoattractant protein-1 (MCP-1) and CpG DNA-induced expression of COX-2, IL-1β, TNF-α, and MCP-1 (Figure 3). Collectively, the results showed that the treatment of peritoneal macrophages with harmine results in the inhibition of the expression of inflammatory mediators induced by multiple TLRs.

### 3.3. Harmine Inhibits the NF-κB Pathway, Independent of IκBα Degradation

**Figure 4:**
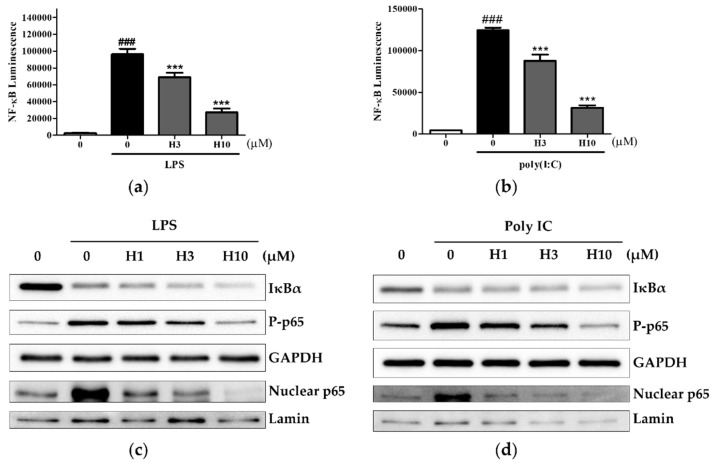
Harmine inhibits NF-κB activity through modulation of p65 phosphorylation during LPS or poly(I:C) activation. (**a**,**b**) RAW 264.7 cells transfected with an NF-κB-dependent reporter gene were cultured with harmine for 12 h and then stimulated with LPS or poly(I:C) for 6 h. NF-κB transcriptional activity was measured using the luciferase assay (n = 3). ### *p* < 0.005 vs. stimulus (–) control, *** *p* < 0.005 vs. stimulus (+) control. (**c**,**d**) Peritoneal macrophages from BALB/c mice were cultured with harmine for 12 h and then stimulated with LPS for 30 min or poly(I:C) for 1 h. Whole cell protein was extracted for analysis of IκBα, phospho-p65, and GAPDH, and nuclear protein was extracted for analysis of p65 and lamin. A representative experiment of three independent experiments is shown.

We investigated how harmine affected the NF-κB signaling pathway induced by TLR4 and TLR3. Previously, Liu et al. showed that harmine, at concentrations ranging from 10 μM to 50 μM, inhibited LPS-induced NF-κB transcriptional activity using a luciferase reporter assay [19]. Using the same assay, high luminescence signals were observed in NF-κB-transfected RAW264.7 cells in the presence of LPS or poly(I:C), indicative of active NF-κB transcription (Figure 4a,b). A lower dose range of harmine was sufficient to inhibit LPS or poly(I:C)-induced NF-κB transcriptional activity. As most NF-κB inhibitors regulate IκBα degradation, we determined whether harmine affected this process. In our preliminary experiments, time exposure to LPS and poly(I:C) required for complete IκBα degradation and nuclear p65 migration was 30 min and 1 h, respectively. Based on these different periods of stimulation, we tested the effect of harmine on IκBα. At 30 min after stimulation with LPS and 1 h after stimulation with poly(I:C), harmine did not inhibit IκBα degradation (Figure 4c,d and Figure S2c,d). The phosphorylation of p65 is another molecular event that can regulate NF-κB activation outcomes. We specifically examined serine 276 phosphorylation of p65, a commonly investigated modification. The amount of LPS or poly(I:C)-induced phosphorylation of p65 was inhibited by the harmine treatment. This paralleled the decrease in the translocation of nuclear p65. Harmine inhibited LPS or poly(I:C)-induced NF-κB activation downstream of IκBα degradation.

### 3.4. Harmine Inhibits AP-1 Activity through Modulation of JNK

**Figure 5:**
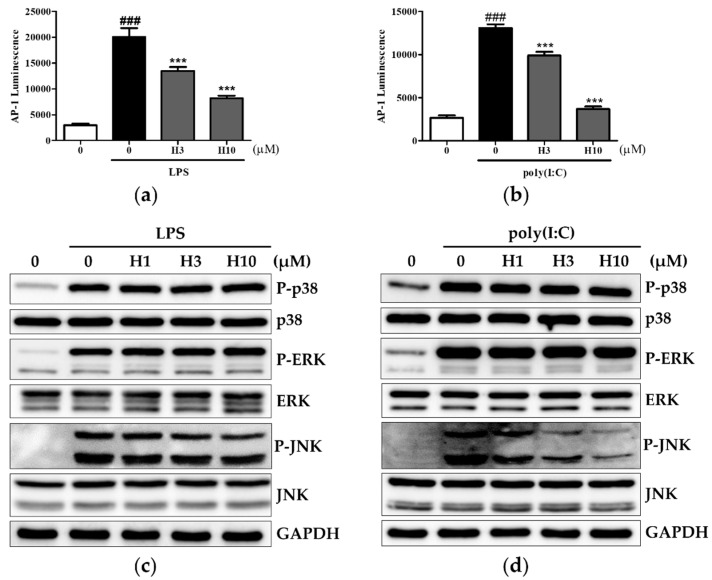
Harmine inhibits AP-1 activity through the modulation of JNK during LPS or poly(I:C) activation. (**a**,**b**) RAW 264.7 cells transfected with an AP-1-dependent reporter gene were cultured with harmine for 12 h and then stimulated with LPS or poly(I:C) for 6 h. AP-1 transcriptional activity was measured using the luciferase assay (n = 3). ### *p* < 0.005 vs. stimulus (–) control, *** *p* < 0.005 vs. stimulus (+) control. (**c**,**d**) Peritoneal macrophages from BALB/c mice were cultured with harmine for 12 h and then stimulated with LPS for 30 min or poly(I:C) for 1 h. Whole cell protein was extracted and analyzed by western blotting. A representative experiment of three independent experiments is shown.

MAPKs such as p38, JNK, and ERK are the major kinases required for AP-1 activation; they also participate in NF-kB activation [21,25]. The influence of harmine on TLR4- and TLR3-induced AP-1 transcriptional activity and MAPK activation was evaluated. In response to LPS or poly(I:C), high luciferase signals were generated in AP-1-transfected RAW264.7 cells and harmine inhibited such increases (Figure 5a,b). Activation of p38, JNK, and ERK was observed in BALB/c macrophages when stimulated with LPS for 30 min or poly(I:C) for 1 h (Figure 5c,d and Figure S2e,f). Among these kinases, harmine attenuated JNK phosphorylation for both stimuli. Thus, the modulation of AP-1 activity by harmine was partially due to its downregulation of JNK activity.

### 3.5. Harmine Inhibits STAT1 Activity

**Figure 6:**
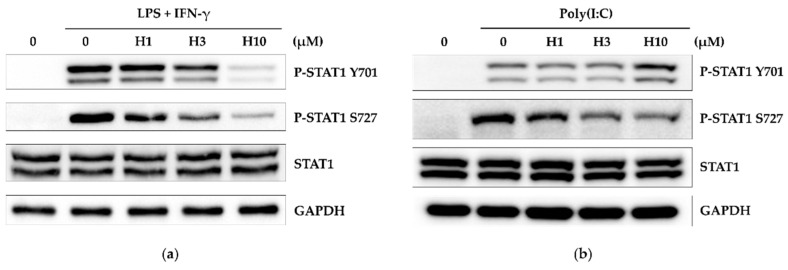
Harmine has a different regulatory effect on STAT1 activation during LPS and poly(I:C) stimulation. Peritoneal macrophages from BALB/c mice were cultured with harmine for 12 h. (**a**) Cells were primed with IFN-γ for 1h and then stimulated with LPS for 2 h. (**b**) Cells were stimulated with poly(I:C) for 2 h. Whole cell protein was extracted and analyzed by western blotting. A representative experiment of three independent experiments is shown.

STAT1 is essential in LPS-induced iNOS expression and acts synergistically with NF-κB for the induction of inflammatory cytokines, including TNF-α [10,26]. As harmine was found to be a potent inhibitor of iNOS, its effect on STAT1 was evaluated. In our experimental conditions, BALB/c macrophages were primed with IFN-γ for 1 h and then stimulated with LPS for 2 h to induce detectable phosphorylation bands at tyrosine 701 and serine 727 (Figure 6a and Figure S2g). Harmine inhibited these two phosphorylation events. As for poly(I:C), treatment with poly(I:C) alone for 2 h resulted in tyrosine and serine phosphorylation, which indicates there was the autocrine production of type I IFN (Figure 6b and Figure S2h). Remarkably, with poly(I:C), serine 727 phosphorylation was attenuated whereas tyrosine 701 phosphorylation was somewhat enhanced. Harmine had a different effect on STAT1 phosphorylation, depending on the stimuli used.

### 3.6. Effect of Harmine on the Liver Inflammatory Response during Endotoxemia

**Figure 7:**
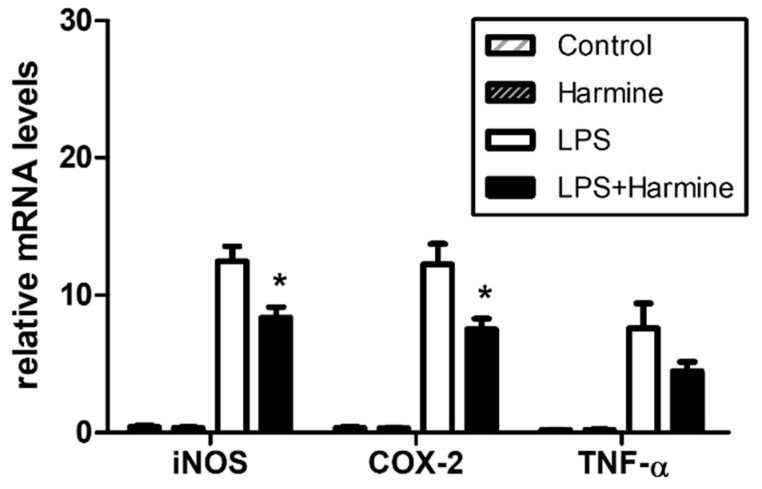
In vivo suppression of inflammatory mediators by harmine. C57BL/6 mice were intraperitoneally(i.p.) injected with saline (white bars) or harmine (black bars) at 30 mg/kg for 4 days and then received an i.p. challenge with LPS (1 mg/kg) for 3 h. The expression of iNOS, COX-2, and TNF-α were analyzed by real-time PCR. Data are the means ± SEM (n = 6). * *p* < 0.05 vs. LPS-treated control group.

Harmine was found to ameliorate the lung and kidney damage induced by the intraperitoneal injection of LPS [19,20]. In this model of endotoxemia, the liver, which contains the largest number of tissue-resident macrophages, called Kupffer cells, in the body, is a major organ that degrades LPS while regulating the spillover of the serum inflammatory mediators [27,28]. We evaluated whether in vivo treatment of harmine affected the liver inflammatory response. The upregulation of iNOS, COX-2, and TNF-α gene expression at 3 h after LPS challenge was observed (Figure 7). Harmine suppressed liver inflammation by inhibiting the expression of the COX-2 and iNOS genes. The suppression of TNF-α was not statistically significant.

## 4. Discussion

Bacterial and viral infections drive researchers to consider natural compounds for the amelioration of microbially induced inflammatory damage. TLR2 and TLR4 are responsible for bacteria-induced inflammation. while TLR3 and TLR9 are involved in the recognition of endocytosed bacteria and viruses. Furthermore, as infection progresses, damaged host cells release endogenous DAMPs, which are detected by TLRs and aggravate the situation [4]. As harmine inhibits the inflammatory expression induced by a broad range of TLR ligands, it can be applied to various infectious inflammatory diseases and their complications.

Notably, we showed that harmine inhibited the TLR4-induced production of IL-12 (IL-12 p70). IL-12 is a heterodimer of p35 and p40 chains, each encoded by separate genes [8]. IL-12 is produced mainly by professional antigen presenting cells such as dendritic cells, macrophages, and monocytes, which present antigens to T cells. CD4 T cells respond to IL-12, differentiating into IFN-γ-secreting Th1 cells [8]. As RAW 264.7 cells do not produce IL-12 p70 owing to their inability to induce p40, the capacity of druggable compounds or natural products to inhibit IL-12 p70 is often neglected [29]. Because Th1 cells produce IFN-γ, which enhances the status of macrophage activation, the inhibition of IL-12 by harmine is a potential anti-inflammatory and immunomodulatory action.

Regarding NF-κB regulation, IκBα degradation and p65 phosphorylation are important steps that anti-inflammatory compounds can interrupt. At lower doses (under 10 μM), harmine did not influence IκBα degradation. Similarly, other natural compounds have shown no effect in IκBα degradation levels, while they still affected p65 phosphorylation [30]. Nonetheless, this is in contrast to the in vivo experiment in which harmine reduced the phosphorylation of IκBα in the kidney damaged by a high dose of LPS (20 mg/kg); this result indicates that harmine may have an affect upstream of IκBα degradation [20]. Such inconsistencies may be due to the dose used or the intensity of stimulus. In support of this, under 20 μM harmine decreased NF-κB transcriptional activity, with little effect on IκBα degradation in Hec-1-A cells infected with herpes simplex virus-2 (HSV-2) (MOI = 1), but inhibited IκBα degradation induced by a higher infectivity of HSV-2 (MOI = 2) [18].

The anti-inflammatory mechanism of harmine may include the modulation of AP-1 activity via the inhibition of JNK during LPS or poly(I:C) activation. AP-1 is a dimeric transcription factor consisting of multiple Jun, Fos, ATF, and Maf members, facilitating the transactivation of NF-κB-specific target genes [21]. When TLRs recruit MyD88 or TRIF, the assembly of IRAK1, IRAK4, and TRAF6 is formed, leading to TAK1 activation, at which point, the NF-κB and MAPK pathways diverge [31]. Because harmine specifically inhibited JNK, not p38, ERK, and IκB kinase, it is likely to influence targets that are downstream of TAK1.

Harmine inhibited tyrosine phosphorylation induced by LPS/IFN-γ, but not by poly(I:C). Rather, it enhanced tyrosine phosphorylation induced by poly(I:C). These disparate results indicate that the inhibitory effect of harmine on STAT1 tyrosine phosphorylation most likely varies, depending on the stimulus type. While JAK is the dominant kinase phosphorylating tyrosine 701, there is JAK independent phosphorylation of tyrosine [32]. The enhanced tyrosine phosphorylation by harmine during poly(I:C) activation suggests that the autocrine generation of type I IFN, which accounts for JAK activity, may increase or that alteration in an unknown tyrosine kinase activity may exist. Unlike tyrosine phosphorylation, harmine inhibited serine phosphorylation induced by LPS/IFN-γ or poly(I:C), indicated that it may interfere with kinases that are specific for serine 727, regardless of the stimulus type. Serine phosphorylation of STAT1 plays a key role in its transcriptional activity [33]. Given the crosstalk between NF-κB and STAT1, inhibition of STAT1 serine phosphorylation by harmine accounts for its overall anti-inflammatory mechanism. Moreover, there is evidence that JNK is associated with IFN-induced STAT1 activation. Inhibiting JNK activity attenuates STAT1 phosphorylation at serine in IFNα-treated monocytic THP-1 cells [34]. The relevance of JNK to such inhibition of STAT1 serine phosphorylation by harmine during TLR activation needs further investigation.

The liver, as the primary organ responsible for the removal of microbes or toxins, including LPS, from the blood and gut, accounts for most of serum inflammatory cytokine production during endotoxemia [27,28]. Harmine administration through the intraperitoneal route suppressed the upregulation of iNOS and COX-2 in the liver during endotoxemia, which indicates that Kupffer cells respond to harmine. In this case, the effects of harmine on Kupffer cells in vivo are assumed to occur in a similar manner to that observed in vitro, as shown here. iNOS catalyzes the generation of nitric oxide (NO), which is microbicidal but can be toxic to host cells when its production is excessive [35]. COX-2, a target of non-steroidal anti-inflammatory drugs, mediates vasodilation and platelet aggregation by producing prostaglandins [36]. Inhibition of iNOS and COX-2 by harmine in vivo offers the potential to be applicable to systemic inflammation. In our analysis, the downregulation of the TNF-α gene at 3 h after LPS challenge was not statistically significant. Because the expression of TNF-α during endotoxemia is transient, earlier measurement may have been required. The suppression of the liver inflammatory response by harmine leads to reduced spillover of LPS and cytokines, contributing to a lower burden on organs that are susceptible to endotoxemia, such as the lungs and kidneys. Further study is required to evaluate its efficacy in a chronic inflammation model.

In this study, we used macrophages from C57BL/6 and BALB/c strains, which are the most common laboratory mice. The function of macrophages differs depending on the tissue microenvironment signals and can be broadly classified into M1 and M2 macrophages. The M1/M2 phenotype is mainly determined by arginine metabolism; M1 macrophages show microbicidal capacity by converting arginine into NO and citrulline whereas M2 macrophages, which can be considered as the default, switches arginine metabolism to ornithine and polyamines [37]. Regarding their macrophage phenotypes, C57BL/6 and BALB/c macrophages can be considered as the M1 and M2 phenotypes, respectively, because BALB/c macrophages are less efficient in NO production compared with C57BL/6 macrophages [22,38]. However, it should be noted that this macrophage dichotomy is an oversimplification; there are intermediate phenotypes that have not yet been completely explored [39]. Certainly, the different immune status of macrophages causes these strains to be susceptible to different microbial infections [38]. Although we showed that harmine can inhibit the activation of the different types of macrophages, appropriate in vivo models are warranted to evaluate whether harmine is effective against the M1 and M2 phenotypes or intermediates.

## 5. Conclusions

This study demonstrates that harmine inhibits the expression of inflammatory mediators induced by multiple TLRs in macrophages by modulating the activation of NF-κB p65, JNK, and STAT1, as well as liver inflammation during endotoxemia. Such findings provide evidence that harmine is effective against inflammation induced by extracellular microbes, including viruses. Knowledge of the precise target of harmine as a kinase inhibitor may make this compound an attractive phytochemical for future drug development.

## Data Availability

Not applicable.

## Supplementary Materials

The following supporting information can be downloaded at: https://www.mdpi.com/article/10.3390/life12122022/s1, Figure S1: Cell viability of harmine. Figure S2: Quantification of protein band obtained from western blotting analysis. Figure S3: Harmine inhibits LPS-induced inflammatory marker genes.
