# Supplementary material for: Harmine Inhibits Multiple TLR-Induced Inflammatory Expression through Modulation of NF-κB p65, JNK, and STAT1
Source: Life (Basel). 2022 Dec 3;12(12):2022. doi: 10.3390/life12122022 (PMC9787735; doi:10.3390/life12122022)
Supplement: Supplementary file 1 [file life-12-02022-s001.zip › life-2013425-supplementary.pdf]

Harmine inhibits multiple TLR-induced inflammatory expression through modulation of NF-κB p65, JNK, and STAT1

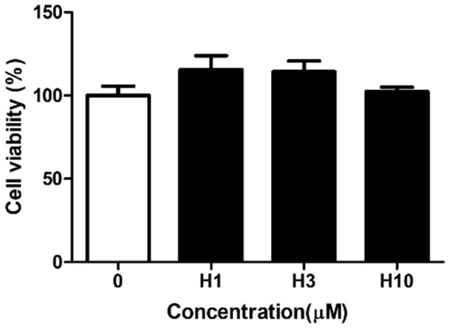

Figure S1. Cell viability of harmine. Peritoneal macrophages were isolated from thioglycollate-injected C57BL/6 mice. Cells (4×10^4 cells) were seeded in 96-well plates overnight and cultured with harmine for 24 h. The cell viability after 24 h was determined by the MTT assay and bars indicate the percentages relative to control cells (0 μM) (n=2).

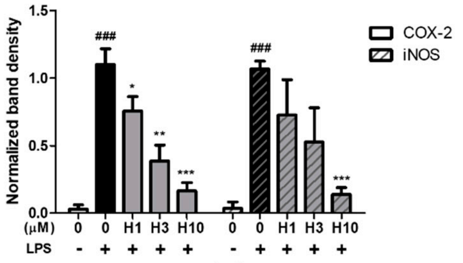

(a)

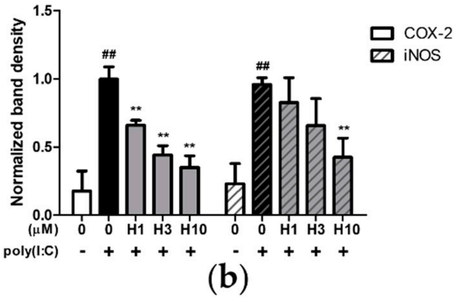

(b)

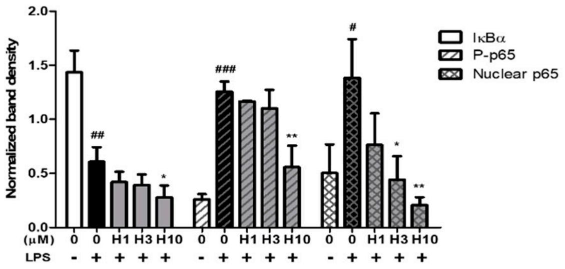

(c)

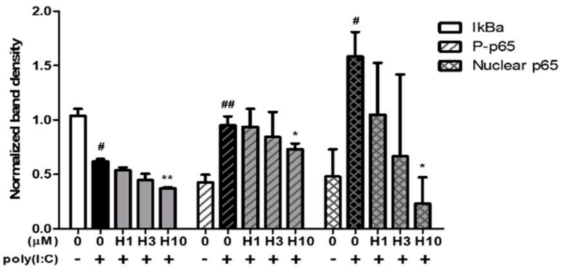

(d)

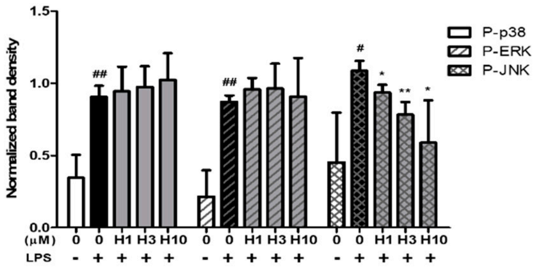

(e)

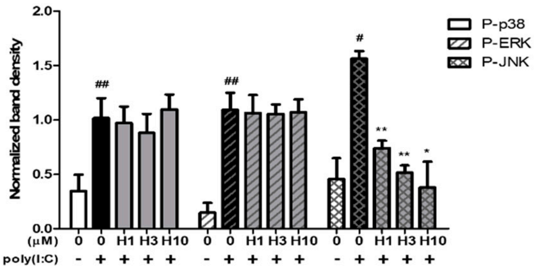

(f)

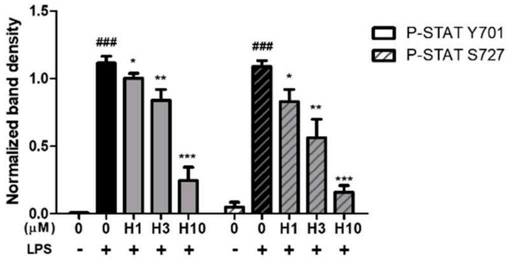

(g)

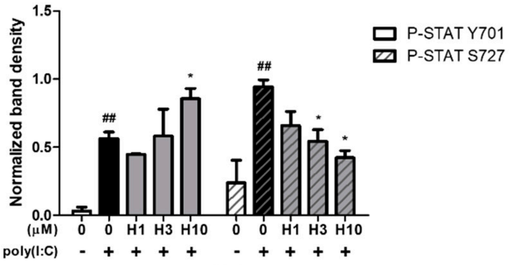

(h)

Figure S2. Quantification of western blots. The band density of each protein was quantified using ImageJ software and normalized to that of GAPDH (n=3). (a) LPS-induced COX-2 and iNOS (Figure 1b). (b) Poly(I:C)-induced COX-2 and iNOS (Figure 2a). (c) LPS-induced IκBα, p-p65, and nuclear p65 (Figure 4c). (d) Poly(I:C)-induced IκBα, p-p65, and nuclear p65 (Figure 4d). (e) LPS-induced p-p38, p-ERK, and p-JNK (Figure 5c). (f)

Poly(I:C)-induced p-p38, p-ERK, and p-JNK (Figure 5d). (g) LPS-induced p-STAT1 Y701 and S727 (Figure 6a). (h) poly(I:C)-induced p-STAT1 Y701 and S727 (Figure 6b). # $p<0.05$, ## $p<0.01$, ### $p<0.005$ vs. stimulus (-) control; * $p<0.05$, ** $p<0.01$, *** $p<0.005$ vs. stimulus (+) control

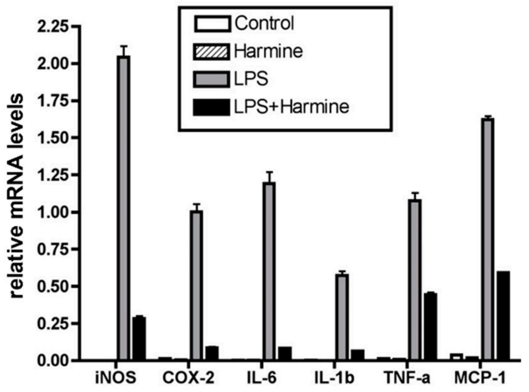

Figure S3. Harmine inhibits LPS-induced inflammatory marker genes. Peritoneal macrophages from C57BL/6 mice were cultured with harmine (10 μM) for 12 h and stimulated with LPS (100 ng/mL) for 6 h. Gene expression was analyzed using real-time RT PCR (n=2).
